# Characterization of Insulin-Like Growth Factor Binding Protein-5 (IGFBP-5) Gene and Its Potential Roles in Ontogenesis in the Pacific Abalone, *Haliotis discus hannai*

**DOI:** 10.3390/biology9080216

**Published:** 2020-08-09

**Authors:** Md. Rajib Sharker, Soo Cheol Kim, Shaharior Hossen, Kang Hee Kho

**Affiliations:** Department of Fisheries Science, College of Fisheries and Ocean Sciences, Chonnam National University, 50 Daehak-ro, Yeosu, Jeonnam 59626, Korea; mrsharker@pstu.ac.bd (M.R.S.); greatguy@chonnam.ac.kr (S.C.K.); 186465@jnu.ac.kr (S.H.)

**Keywords:** *Haliotis discus hannai*, IGFBP-5, neural ganglia, mRNA expression, in situ hybridization

## Abstract

Insulin-like growth factor binding protein family is known to be involved in regulating biological actions of insulin-like growth factors (IGFs). In the present study, a full-length cDNA encoding the IGFBP-5 gene was cloned and characterized from the cerebral ganglion of *Haliotis discus hannai*. The 921-bp full-length sequence of Hdh IGFBP-5 cDNA transcript had an open reading frame of 411 bp encoding a predicted polypeptide of 136 amino acids, sharing high sequence identities with IGFBP-5 of *H. diversicolor*. The deduced Hdh IGFBP-5 protein contained a putative transmembrane domain (13-35 aa) in the N-terminal region. It also possessed a signature domain of IGFBP protein family (IB domain, 45-120 aa). Six cysteine residues (Cys-47, Cys-55, Cys-73, Cys-85, Cys-98, and Cys-118) in this cloned sequence could potentially form an intrachain disulfide bond. Phylogenetic analysis indicated that the Hdh IGFBP-5 gene was robustly clustered with IGFBP-5 of *H. diversicolor*. Tissue distribution analysis based on qPCR assay showed that Hdh IGFBP-5 was widely expressed in all examined tissues, with significantly (*p* < 0.05) higher expression in the cerebral ganglion. In male and female gametogenetic cycles, Hdh IGFBP-5 mRNA was expressed at all stages, showing significantly higher level at ripening stage. The expression level of Hdh IGFBP-5 mRNA was significantly higher in the polar body stage than in other ontogenic stages. In situ hybridization revealed that Hdh IGFBP-5 mRNA was present in the neurosecretory cells of the cerebral ganglion. This is the first study describing IGFBP-5 in *H. discus hannai* that might be synthesized in the neural ganglia. Our results demonstrate Hdh IGFBP-5 is involved in regulating ontogenic development and reproductive regulation of *H. discus hannai*.

## 1. Introduction

Insulin-like growth factors (IGFs) are evolutionary conserved signaling proteins ubiquitously distributed in animals including invertebrates [1]. These polypeptides play critical roles in neuroendocrine regulation of growth, development, metabolism, reproduction, and aging in vertebrates and invertebrates [2,3,4,5,6,7]. In vertebrates, the IGF system consists of two IGF ligands (IGF-I and IGF-II), two IGF receptors (IGF-IR and IGF-IIR), and a family of IGF binding proteins (IGFBPs) [8,9]. The IGF signaling system plays a pivotal role in regulating embryonic and larval development as well as in maintaining homeostasis of adults in various species [10]. IGFBPs are key regulators of IGF activity to regulate the neuroendocrine system. IGF ligands can bind to insulin/IGF family of cell surface receptors and activate their intrinsic tyrosine kinase domain activities, thus regulating a number of biological responses [11]. During this biological process, IGFBPs can interact and transport IGFs as carriers and regulate the physiological mechanism of IGFs by mediating their interactions with IGF receptors [11]. Six IGFBP members designated as (IGFBP1-6) have been cloned and characterized in mammalian vertebrates, which bind IGFs with high affinity [10,12,13]. They are synthesized in different levels from various cells and tissues of vertebrates, suggesting a fundamental role in the regulation of IGF actions [13]. The architecture of IGFBPs consists of three domains: N-terminal and C-terminal domains, which both contribute to its high-affinity IGF binding, and the variable central linker domain that is believed to be important for maintaining structural integrity [14]. Although different forms of IGFBPs share considerable sequence similarities, each IGFBP type possesses distinct properties and performs stimulatory or inhibitory functions in different IGF-mediated biological mechanisms [15,16]. The differences in their functions are influenced by post-transcriptional modification such as N-linked glycosylation, phosphorylation for protein kinase A or C, proteolysis, and cell surface associations [17].

IGFBP-5 is the most conserved IGFBP and has been considered as a key regulator during the development of essential cell lineages and neural cells [18,19,20]. This protein plays a significant role in growth, development, and reproduction [21]. IGFBP-5 can exert multiple biological functions by binding IGF with high affinity through an N-terminal motif [22]. It is known to modulate IGF-I activity in neural tissues [21]. It has been reported that IGFBP-5 plays a role in various aspects of teleost biology, including ionic homeostasis and IGF regulation in zebrafish [23], stickleback [24], and Atlantic salmon [16]. IGFBP-5 also appears to be involved in the regulation of biomineralization in the mantle and pearl sac of pearl oysters [25].

Abalone, a top-priced marine bioresource, is widely distributed throughout temperate and tropical coastal regions [26]. Of various *Haliotis* species, *H. discus hannai* has a high commercial value in southern coasts of China, Japan, Taiwan, and the Korean peninsula owing to the presence of bioactive molecules compounds with antioxidant, antithrombotic, anti-inflammatory, antimicrobial, and anticancer activities in this species [27]. Many studies on IGFBPs have been performed in vertebrate species including *Homo sapiens* [28], *Mus musculus* [29], *Oncorhynchus mykiss* [30], and *Danio rerio* [31]. However, little information is available about IGFBPs in mollusks. A molluscan IGFBP-7 gene cloned from *H. diversicolor* has been suggested to be involved in a function associated with pathogenic infections in adults [32]. An IGFBP gene cloned and characterized from Yesso scallop (*Patinopecten yessoensis*) [9] has been suggested to have a crucial role in scallop growth and development [9]. A full-length cDNA encoding IGFBP-5 gene was isolated and characterized from the *Pinctada fucata* (Pf-IGFBP-5), and the results indicated that this gene may be involved in shell formation of bivalve mollusks [25]. However, there is no report on cDNA cloning, characterization, and expression analysis of the IGFBP-5 gene in *H. discus hannai*. To understand the role of IGFBP-5 in growth, reproduction, and metamorphosis of abalone, we cloned and characterized the full-length cDNA of the IGFBP-5 gene and examined the expression patterns during the gametogenetic cycle and ontogenetic development, and in adult tissues. Furthermore, the cellular localization of IGFBP-5 was determined using in situ hybridization.

## 2. Materials and Methods

### 2.1. Experimental Animals and Sample Collection

Adult male and female abalone were obtained from Jindo Island (South Korea) and transferred to the laboratory in the Department of Fisheries Science, Chonnam National University. Various tissues including pleuropedal ganglion, cerebral ganglion, branchial ganglion, adductor muscle, ovary, testis, and mantle were collected following anesthetization on ice. Different embryonic and larval developmental stages (polar body, 2-cell, 8-cell, 16-cell, morula, blastula, trochophore, and veliger) were collected and frozen immediately in liquid nitrogen before storage at −80 °C for subsequent experiment.

For cryosection, cerebral ganglion tissues were washed in phosphate-buffered saline (PBS; pH 7.4) and immersion fixed in 4% paraformaldehyde (PFA) overnight. A brief procedure of cryosection preparation was described previously [33,34].

Collection and handling of *H. discus hannai* were performed in compliance with the guidelines and approval of Institutional Animal Care and Use Committee of Chonnam National University (CNU IACUC) in accordance with Article 14th of the Korean Animal Protection Law of the Korean government. No specific permission was required to perform the experiment with abalone in South Korea.

### 2.2. RNA Isolation and cDNA Synthesis

Total RNA was isolated from each tissue and different developmental stages of Pacific abalone using an RNeasy mini kit (Qiagen, Hilden, Germany) and treated with RNase-free DNase (Promega, Madison, WI, USA) to remove genomic DNA. RNA quality was assessed by electrophoresis on a 1% (*w*/*v*) agarose gel, and concentration was determined with a spectrophotometer (NanoDrop^®^ NP 1000 spectrophotometer, Thermo Fisher Scientific, Waltham, MA, USA). Total RNA (1 µg) was reverse transcribed to cDNA using Superscript^®^ III First-Strand synthesis kit (Invitrogen, Carlsbad, CA, USA) according to the manufacturer’s protocol for further PCR analysis.

### 2.3. Cloning of IGFBP-5 from Pacific Abalone

Molecular cloning was performed to identify the IGFBP-5 gene using reverse transcription (RT) primers (forward: 5′-CTTCGCTGACAACGATGTCC-3′ and reverse: 5′-GTCACTTCTGCGCATGTCTG-3′). These primers were designed based on known *H. diversicolor* IGFBP-5 cDNA sequence (GenBank accession no. JX854538.1). PCR amplification was performed in a 20 μL reaction volume, containing 1 μL (20 pmol) each of forward and reverse primers, 4 μL of 5× Phusion HF buffer (1×), 2 μL of dNTP (200 μM), 0.5 μL of 1 U Phusion DNA polymerase, 10.5 μL sterile distilled water (dH_2_O), and 1 μL of the synthesized cDNA from the cerebral ganglion as a template. PCR cycling profile was as follows: 5 min at 94 °C, followed by 35 cycles of 30 s at 94 °C, 1 min at 58 °C, 30 s at 72 °C, with a final dissociation step of 7 min at 72 °C. The amplified PCR product was separated on 1.2% agarose gel electrophoresis, purified using a gel extraction kit (Promega), and cloned into pTOP Blunt V2 vector (Enzynomics, Daejeon, Korea). Positive clones were sequenced using Macrogen Online Sequencing System (Macrogen, Seoul, Korea). To obtain full-length sequence, 5′, and 3′ rapid amplification of cDNA ends (RACE) was performed using first-strand cDNA from the cerebral ganglion as a template with a universal primer mix (UPM) of 5′-CTAATACGACTCACTATAGGGCAAGCAGTGGTATCAACGCAGAGT-3′ and gene-specific primer sequences (GSPs) that included a 15-bp overlap with the 5′-end of GSP sequence (antisense primer: 5′-GATTACGCCAAGCTTGCACACACCTCTACCCAGCAGCAAG-3′, sense primer: 5′-GATTACGCCAAGCTTAAACTTCACCACGTGGCTTCGATTTCCC-3′). Touchdown PCRs of 30 cycles and 35 cycles were conducted for 3-RACE and 5-RACE, respectively, following the kit manual. Purified RACE PCR products were cloned into a linearized pRACE vector (Clontech Laboratories, Inc., Mountain View, CA, USA) followed by transformation into Stellar Competent Cells for amplification and sequencing with a Macrogen Online Sequencing System (Macrogen, Seoul, Korea). Sequenced RACE products were then combined by overlapping with the initial fragment.

### 2.4. Sequence Analysis

A protein homology study was conducted with Basic Local Alignment Search Tool (BLAST) algorithm at NCBI (http://www.ncbi.nlm.nih.gov/BLAST/) for similarity to known gene. Protein transmembrane domain features were predicted with SMART tool (http://smart.embl-heidelberg.de/). NetPhosK 3.0 server was used to predict serine/threonine phosphorylation sites. The bonding state of cysteine in the protein sequence was determined using CYSPRED [35]. Physical and chemical properties of the protein were predicted using ProtParam (http://expasy.org/tools/protparam.html). Subcellular localization was determined with Protcomp (http://linux1.softberry.com/berry.phtml). Multiple alignments of IGFBP-5 proteins of *H. discus hannai* and other species were accomplished with Clustal Omega [36].

### 2.5. Phylogenetic Analysis

To construct the phylogenetic tree, IGFBP protein sequences from invertebrates and vertebrates were retrieved from the NCBI database and aligned using Clustal Omega [36]. The tree was generated with MEGA software (version 7) using the neighbor-joining algorithm with 1000 bootstrapping replicates [37].

### 2.6. Homology Modeling of IGFBP-5 in Pacific Abalone

Three-dimensional (3D) homology modeling of Pacific abalone IGFBP-5 was performed using I-TASSER server [38]. Chimera software (https://www.cgl.ucsf.edu/chimera/) was used to visualize and analyze the predicted 3D structure of IGFBP-5. ProQ (http://www.sbc.su.se/~bjornw/ProQ/ProQ.html) and ERRAT (http://services.mbi.ucla.edu/ERRAT) tools were used to check the stereo-chemical quality of the predicted model.

### 2.7. Semi-Quantitative Reverse Transcription-Polymerase Chain Reaction (RT-PCR)

For semi-quantitative RT-PCR expression analysis, sequence-specific primers (forward: 5′-CGCGAAACGATGCAGCACATGT-3′ and reverse: 5′-TCCACACCTATGCACACACC-3′) were used. Primers (forward: 5-TGTCCGTTTCACCAACAAGG-3′ and reverse: 5-CAGTGTGCAGCCGCATAGTT-3′) designed from *H. discus hannai* ribosomal protein L-5 (RPL-5, JX002679.1) gene, was used as an internal control based on its expression stability [39]. PCR was performed in a 20 μL reaction volume containing 1 μL cDNA template from the pleuropedal ganglion, cerebral ganglion, branchial ganglion, adductor muscle, testis, ovary, and mantle, 1 μL (10 pmol) of each forward and reverse primer, 10 μL of Prime Taq Premix (2×) (GENETBIO), and 7 μL of sterilized distilled water (dH_2_O). PCR cycling conditions were similar to those as described previously.

### 2.8. Quantitative Real-Time PCR (qRT-PCR) Analysis

A quantitative PCR was performed to determine expression patterns of IGFBP-5 mRNA in different tissues using 2× qPCRBIO SyGreen Mix Lo-Rox kit (PCR Biosystems, Ltd., London, UK). PCR was performed in a 20 µL reaction volume containing 1 μL cDNA template of each tissue, 1 μL (10 pmol) of each forward and reverse primer, 10 μL SyGreen Mix, and 7 μL PCR-grade water. PCR cycling program included a preincubation step at 94 °C for 5 min followed by three-step amplification at 94 °C for 30 s, 60 °C for 1 min, and 72 °C for 30 s for 40 cycles. Three biological replicates (N = 3) were performed for each sample. The melting temperature was 95 °C for 10 s, 65 °C for 1 min, and 97 °C for 1 s used as a default setting. For quantification, the fluorescence reading was documented at the last step of each cycle. Relative mRNA levels of IGFBP-5 were analyzed using the 2^−ΔΔct^ method [40].

To investigate the involvement of Hdh IGFBP-5 in the gametogenetic cycle, expression levels of Hdh IGFBP-5 mRNA in gonadal tissues at different reproductive stages were determined using qRT-PCR assay. Reproductive stages of gonads were classified according to our previous study [41]. cDNAs (1 µL) of testis and ovary at different stages were used as templates for qRT-PCR as described above.

Expression levels of the Hdh IGFBP-5 gene in different developmental stages were determined by qPCR. One microliter of cDNA template from different embryonic and larval stages (polar body, 2-cell, 8-cell, 16-cell, blastula, trochophore, and veliger) was used for qPCR assay.

### 2.9. In Situ Hybridization (ISH)

Distribution of IGFBP-5 mRNA in the cerebral ganglion was analyzed using in situ hybridization with DIG-labeled antisense and sense RNA probes. Primers (forward: 5′-GGTGCTGTCTTCGCTGACAA-3′ and reverse: 5′-CCAGTCTTCTATATGGACAGT-3′) were designed from IGFBP-5 nucleotide sequence, producing 548-bp PCR product. Amplified products were cloned into pGEM-T Easy vector (Enzynomics, Daejeon, Korea) and recombinant plasmids were used as template to synthesize antisense and sense RNA probes by in vitro transcription following a previous method [41,42]. The cerebral ganglion tissue sections were prehybridized with hybridization buffer and yeast total RNA (50 μL) for 2 h, followed by overnight hybridization with DIG-labeled antisense or sense RNA probe at 65 °C. Tissue sections were then washed and blocked with 10% calf serum at room temperature for 1 h. Subsequently, these sections were treated with alkaline phosphatase-conjugated anti-digoxigenin-Ap-Fab fragments antibody (diluted 1:500 in blocking solution [Roche]) overnight at 4 °C to detect the hybridization signal. Finally, sections were treated with substrate BCIP/NBT (Roche) and incubated in a dark and humid chamber for at least 1 h to observe the color. After satisfactory color development, sections were viewed and photographed using a stereo microscope (SMZ1500, Nikon, Tokyo, Japan).

### 2.10. Nuclear Fast Red Counterstain

Antisense probe hybridized ISH slides of the cerebral ganglion were counterstained using nuclear fast red (Sigma-Aldrich, St. Louis, MO, USA) following the protocol described by Sharker et al. [33]. After staining, slides were dehydrated, mounted with Permount mounting medium, hardened, and cleaned for examination under a microscope.

### 2.11. Statistical Analysis

Statistical analysis was performed with one-way analysis of variance (ANOVA), followed by Tukey’s multiple comparisons using SPSS (version 16.0). Differences were considered statistically significant at *p* < 0.05.

## 3. Results

### 3.1. Cloning and Characterization of IGFBP-5

**Figure 1:**
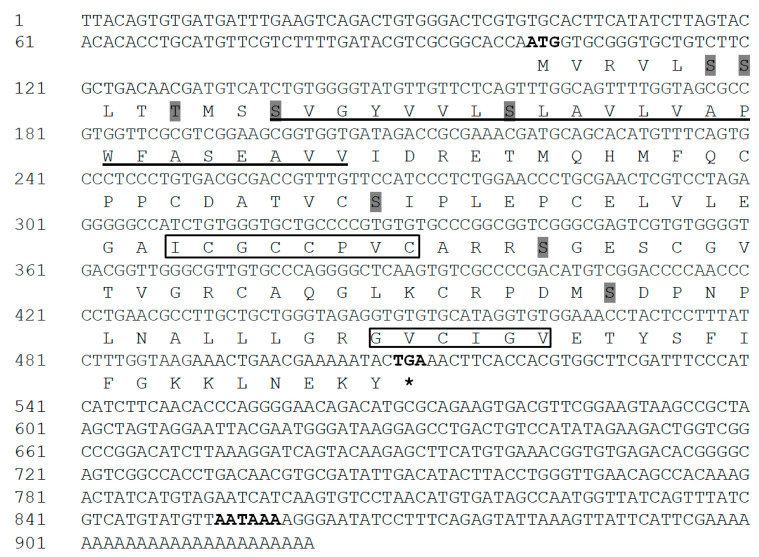
Full-length cDNA and deduced amino acid sequences of Hdh IGFBP-5. Initiation and termination codons (asterisks), putative polyadenylation signals are shown by bold font. The transmembrane region is underlined. Potential phosphorylation sites are marked with shadow. Characteristic motifs (XCGCCXXC and GVCXXV) are denoted by black boxes.

The full-length IGFBP-5 cDNA sequence was obtained from the cerebral ganglion of *H. discus hannai* and referred to as Hdh IGFBP-5. The sequence was deposited into the GenBank database with an accession number of MT345604. The 921-bp cDNA transcript of IGFBP-5 contained an open reading frame (ORF) of 411 bp, including a 100-bp 5′-untranslated region (UTR) and a 410-bp 3′-UTR with a canonical polyadenylation signal motif (AATAAA) located 38 bp upstream of the poly-A tail (Figure 1). The deduced amino acid sequence of 136 residues from its ORF showed that the cloned IGFBP-5 protein shared 81% and 49% sequence identities with *H. diversicolor* and *Pomacea canaliculata* IGFBP-5, respectively. It also exhibited 47%, 46%, and 44% sequence identities with *Homo sapiens*, *Ctenopharyngodon idella*, and *Danio rerio* IGFBP-5 protein, respectively. The predicted molecular weight and isoelectric point (pI) of the cloned sequence was 14.58 kDa and 5.77, respectively. The Hdh IGFBP-5 might be a membrane-bound extracellular secretory protein, based on subcellular localization prediction with Protcomp. Based on sequence analysis, Hdh IGFBP-5 has a putative transmembrane domain (13-35 aa) in the N-terminal region. It possesses an IB domain (45-120 aa), a signature domain of the IGFBP protein family. The cloned sequence contained eight potential sites for phosphorylation by protein kinase A or C. Six cysteine residues (Cys-47, Cys-62, Cys-73, Cys-85, Cys-98, and Cys-118) were likely to form intramolecular disulfide bond.

**Figure 2:**
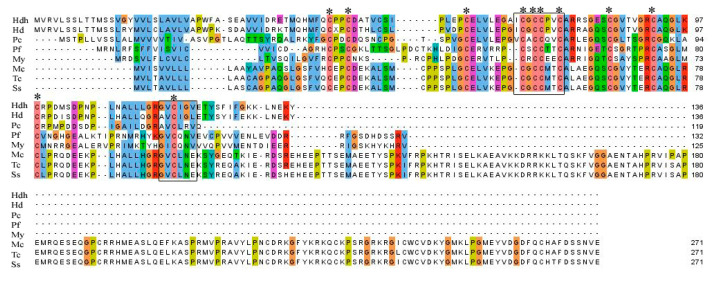
Multiple sequence alignment of amino acid sequences of Hdh IGFBP-5 with representative vertebrate and invertebrate homologs. Conserved motifs are indicated by black boxes. Conserved cysteine residues are marked with asterisks. Hdh–*H. discus hannai*, Hd–*H. diversicolor* (AFY98847.1), Pc–*Pomacea canaliculata* (XP_025085513.1), Pf–*Pinctada fucata* (ALJ32263.1), My–*Mizuhopecten yessoensis* (AHF95042.1), Mc–*Mastomys coucha* (XP_031223763.1), Tc–*Tupaia chinensis* (XP_006157508.1), Ss–*Suricata suricatta* (XP_029789733.1).

Amino acid sequence alignment of IGFBP-5 homologs from different vertebrate and invertebrate species is shown in Figure 2. A relatively high level of amino acid identity was found in the IB domain of the protein. Analysis of conserved domains revealed the signature amino acid motifs of XCGCCXXC and GVCXXV were also well conserved among different species of IGFBP-5 protein.

**Figure 3:**
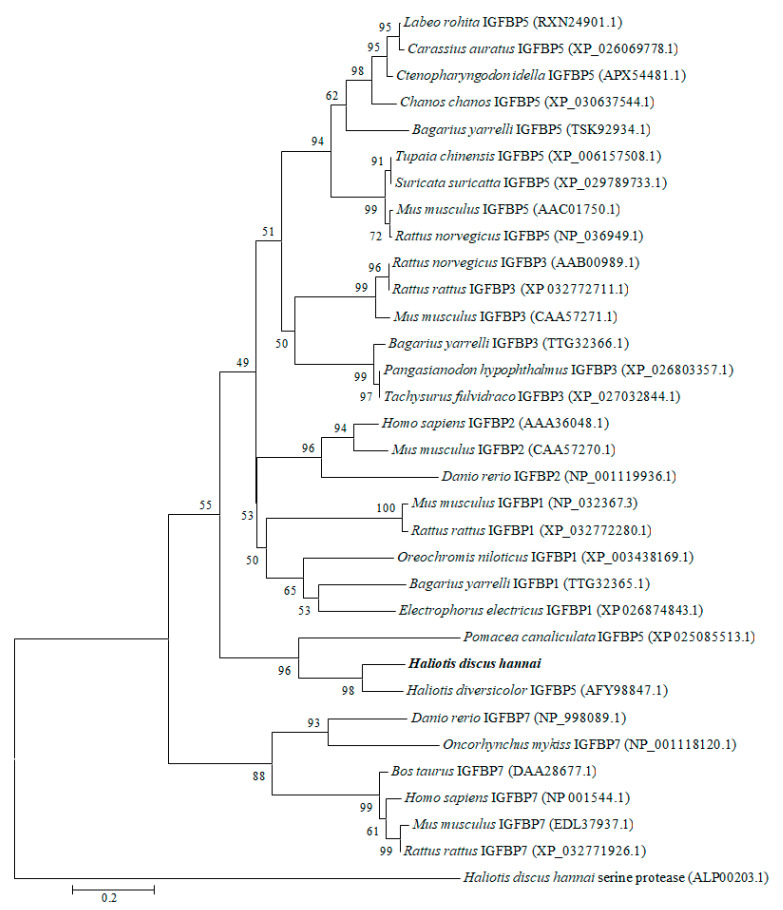
Phylogenetic tree was constructed based on amino acid sequences of IGFBP-5 from vertebrates and invertebrates. Phylogenetic tree analysis was performed using a neighbor-joining method with 1000 bootstrap replicates. The scale bar indicates an evolutionary distance of 0.2 amino acid divergence per site. Hdh IGFBP-5 in this study is highlighted in bold font. GenBank accession numbers are shown in parentheses.

To explore the evolutionary relationships, a phylogenetic tree was constructed with IGFBP proteins of molluscan and selected vertebrates using the neighbor-joining method (Figure 3). The results revealed that molluscan IGFBPs were grouped into one independent clade. Our cloned IGFBP genes of *H. discus hannai* were phylogenetically clustered with IGFBP-5 of *H. diversicolor* with a bootstrap value 98%, indicating that the evolution rule of IGFBP-5 in *H. discus hannai* was in accord with that of *H. diversicolor.* As an outgroup, *H. discus hannai* serine protease was used.

**Figure 4:**
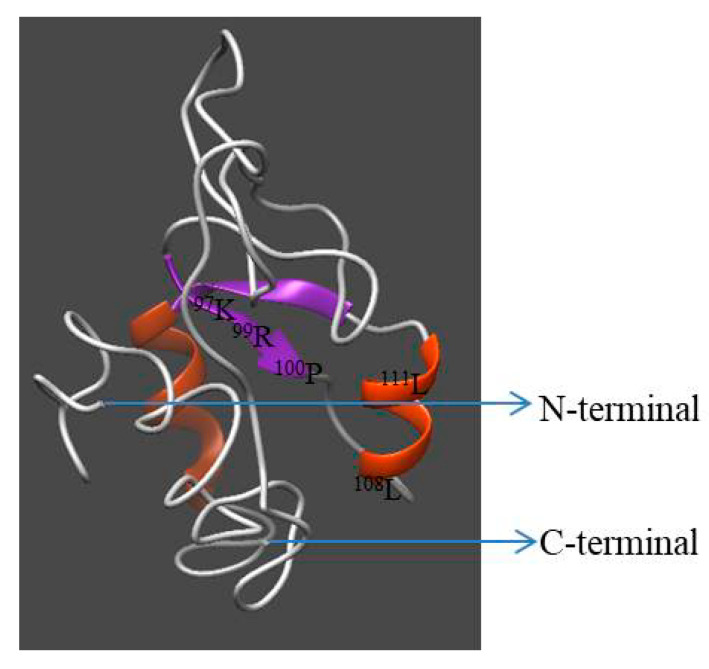
Predicted three-dimensional model of Hdh IGFBP-5. N- and C-termini are marked with arrows. The structure was generated using I-TASSER with a C-score of 2 to 4.

Homology modeling of Hdh IGFBP-5 was generated using the crystal structure of human IGFBP-5 (1H59) with a resolution of 2.1 Å as a template. The 3D structure displayed three-domain organization: the N-terminal and C-terminal domains were α-helix, while the middle domain consisted of antiparallel β-sheets (Figure 4). Seventeen and eight amino acids were involved in the α-helix and β-sheet, respectively. Five ligand binding site residues at positions ^97^K, ^99^R, ^100^P, ^108^L, and ^111^L were detected in this model. ProQ and ERRAT tools were used to validate the stereo-chemical quality of the predicted model. Validation results revealed that the LG score was 2.703 (a value >2.5 indicating a very good model), indicating that the predicted model was very good. Its ERRAT quality factor was 93.43%. These results showed that the predicted 3D structure of Hdh IGFBP-5 amino acid residue was in a favorable position.

### 3.2. Expression Analysis of Hdh IGFBP-5

**Figure 5:**
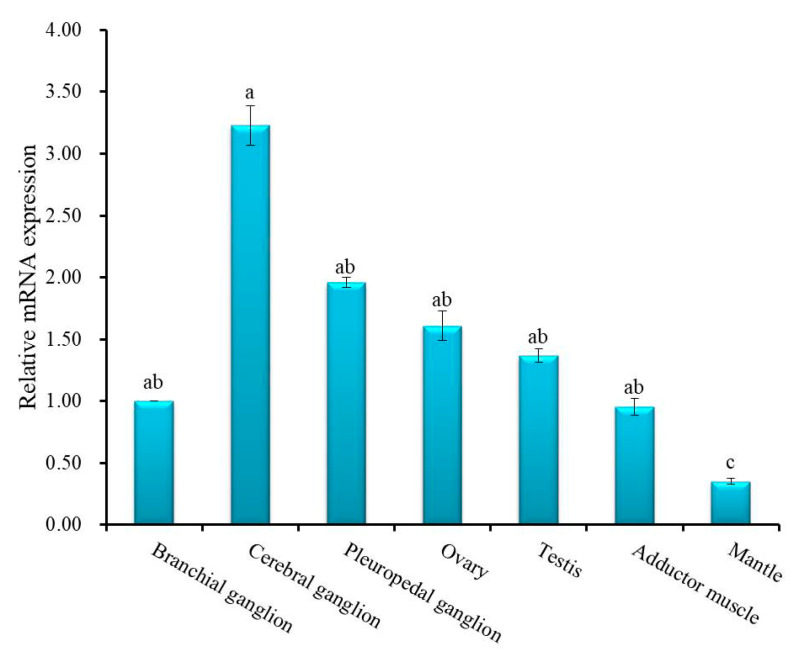
Relative mRNA expression level of Hdh IGFBP-5 (means ± SD, *N* = 3) in various tissues of *H. discus hannai*. Data were compared with relative values in branchial ganglion (1). Different letters indicate significant differences from each other (*p* < 0.05). Significantly higher expression of cerebral ganglion is indicated by a. The expression among branchial ganglion, pleuropedal ganglion, ovary, testis, and adductor muscle are not differ significantly, indicated by ab. Significantly lower expression was found in the mantle tissue, which is indicated by c.

Quantitative real-time polymerase chain reaction (qRT-PCR) was employed to quantify relative mRNA expression of Hdh IGFBP-5 in different tissues (Figure 5). Significantly (*p* < 0.05) higher expression was found in the cerebral ganglion than in other examined tissues. There were no significant differences in the expression of Hdh IGFBP-5 in pleuropedal ganglion, branchial ganglion, testis, ovary, and adductor muscle. Significantly (*p* < 0.05) lower expression was found in the mantle. Supporting data were also found from semi-quantitative RT-PCR expression analysis (Figure S1).

**Figure 6:**
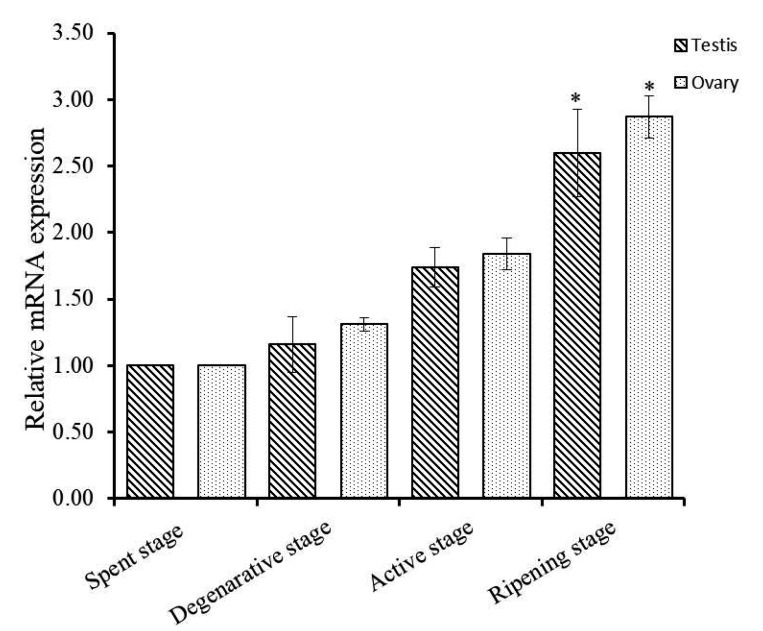
Expression levels of Hdh IGFBP-5 mRNA in *H. discus hannai* gonad at different gametogenetic stages. The expression level in the gonad at spent stage is set as 1 to calibrate expression levels in the gonad at other stages. Asterisks indicate significant differences (*p* < 0.05) in ripening stage than other stages.

To explore the functions of Hdh IGFBP-5 in gonadal development, qRT-PCR assay was conducted to detect the expression pattern of Hdh IGFBP-5 mRNA transcript at different gametogenesis stages. In male and female gametogenetic stages, Hdh IGFBP-5 mRNA was expressed moderately in the spent stage, degenerative stage, and active stage. However, significantly higher expression was found in the ripening stage than in other stages (Figure 6).

**Figure 7:**
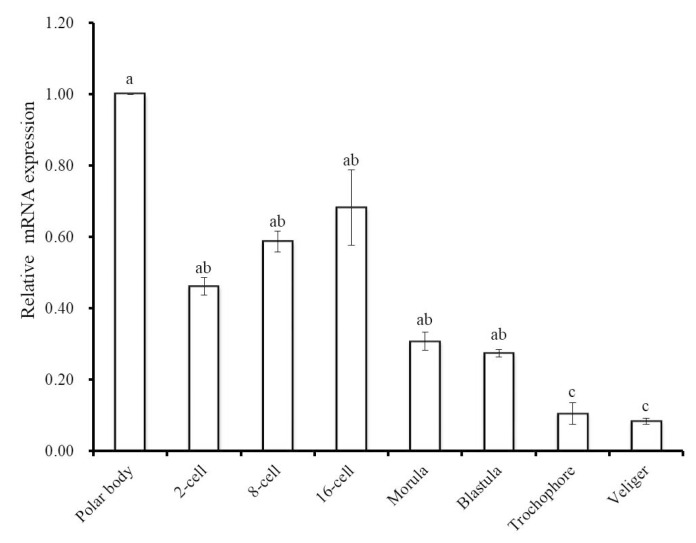
Expression levels of Hdh IGFBP-5 mRNA (means ± SD, *N* = 3) in different embryonic and larval stages of the *H. discus hannai*. Expression in all stages is normalized to expression in the polar body stage. Means not sharing the same letters are significantly different. Significantly higher expression was found in the polar body stage, which is indicated by a. No significant differences were observed in 2-cell, 8-cell, 16-cell, morula, and blastula stages, indicated by ab. c letter denote that there are no significant differences between trochophore and veliger larval stages.

To gain further insight into the involvement of Hdh IGFBP-5 during ontogenesis, qRT-PCR assay was performed to determine the mRNA expression of Hdh IGFBP-5 in different developmental stages (Figure 7). Hdh IGFBP-5 transcript was expressed in all developmental stages with the significantly highest level in the polar body stage. Significantly lower expression was observed in the trochophore and veliger larval stages.

**Figure 8:**
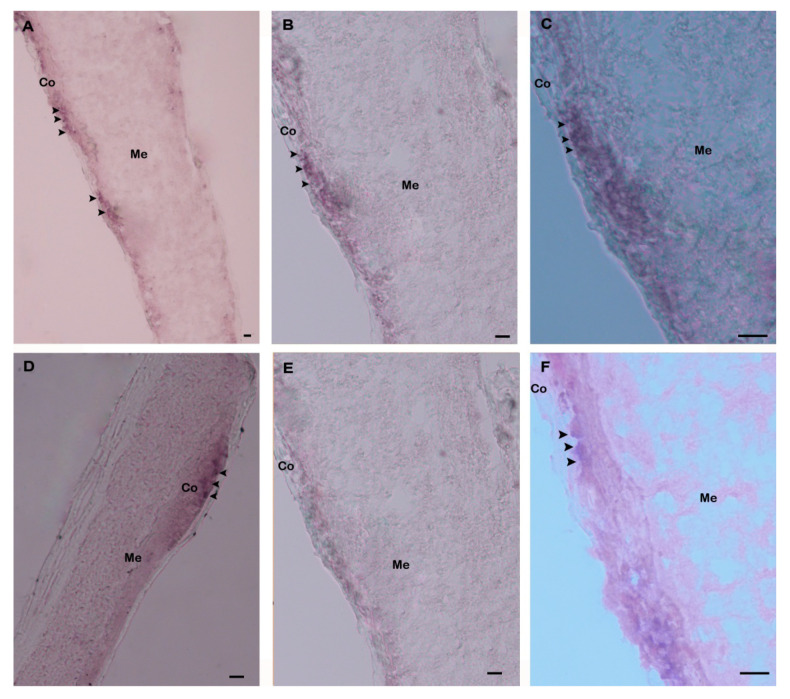
Expression of Hdh IGFBP-5 mRNA in the cerebral ganglion demonstrated by in situ hybridization. (**A**) Hybridization signals with an antisense probe are detected in neurosecretory cells (arrows) of the cortex region; (**B**) medium magnification of A; (**C**) high magnification showing hybridized Hdh IGFBP-5 mRNA in neurosecretory cells; (**D**) medium magnification showing hybridization signal in other parts of the cortex region; (**E**) no hybridization signal is detected after hybridizing with Hdh IGFBP-5 mRNA sense probe; (**F**) fast red counterstaining exhibiting hybridized neurosecretory cells. Co–Cortex; Me–Medullae. Scale bars, 100 μm.

Spatial expression and distribution of Hdh IGFBP-5 mRNA in the cerebral ganglia section were determined using in situ hybridization with a DIG-labeled antisense probe. Positive hybridization signals were observed in the cortex region of the cerebral ganglia section (Figure 8A–D). No hybridization signals were detected in the negative control section due to absence of an antisense probe in the hybridization mix during incubation (Figure 8E). The positive signal was likely to be localized in neurosecretory cells of the cortex region as revealed by fast red counter staining (Figure 8F).

## 4. Discussion

IGFBPs are evolutionary conserved N-terminal cysteine-rich extracellular proteins that play an indispensable role in regulating different physiological processes of vertebrates and invertebrates. IGFBPs are well characterized in mammals. In invertebrate species, several members of the IGFBP superfamily have been reported, including IGFBP-7 in fruit fly *Drosophila* melanogaster [43], Sa-IGFBP-7 in small abalone *Haliotis diversicolor* [32], Py-IGFBP in scallop *Patinopecten yessoensis* [9], Cq-IGFBP in red claw crayfish *Cherax quadricarinatus* [44], Sv-IGFBP in Eastern rock lobster *Sagmariasus verreauxi* [45], and Mn-IGFBP in oriental river prawn *Macrobrachium nipponense* [46]. The IGFBP-5 gene was cloned and characterized from Pearl oyster *Pinctada fucata* and referred to as Pf-IGFBP-5 [25]. To date, cloning and characterization of IGFBP-5 from *H. discus hannai* have not been published yet. In the present study, the complete cDNA sequence of IGFBP-5 was isolated. It encoded a putative protein of 136 amino acid residues. The structural profile of this cloned sequence contained several characteristic features of the IGFBP family, including a transmembrane segment, phosphorylation sites, and an IB domain in the N-terminal region. These observations are in good agreement with the previous report [47,48]. Potential serine/threonine phosphorylation sites in this cloned sequence might play a crucial role in cell signaling [49]. Six cysteine residues could potentially form an intracellular disulfide bridge, suggesting that they might be crucial for three-dimensional conformation of IGFBP disulfide bonds [50] and for IGF binding [51,52]. A BLAST search indicated that this protein shared high similarities with IGFBP-5 of other invertebrate species.

IGFBPs are characterized by conserved motifs XCGCCXXC and GVCXXV in N-terminal and C-terminal domains, respectively [8,53]. Multiple sequence alignment displayed that these typical IGFBP motifs were well conserved in this sequence. The signature amino acid motif XCGCCXXC is considered to play a crucial role in interactions with IGFs [8,54].

The constructed phylogenetic tree demonstrated that the IGFBP of *H. discus hannai* was robustly aligned with *H. diversicolor* IGFBP-5 (Figure 3). Similarly, Zhang et al. [55] have reported that the Pf- IGFBP of *P. fucata* is clustered with IGFBP-5 of yesso scallop. Based on its structural features and high conservation of IGFBP motif, we could infer that the cloned sequence is a potential member of the IGFBP superfamily and designated as Hdh IGFBP-5.

The 3D structure of the Hdh IGFBP-5 protein was determined based on its amino acid sequence using the crystal structure of human IGFBP-5 (1H59) as a template (Figure 4). Evaluation results confirmed structural conservation of the deduced IGFBP-5 protein. Results also showed that its amino acid residues were in favorable positions.

Relative mRNA expression levels of Hdh IGFBP-5 in different tissues were examined using qRT-PCR. Hdh IGFBP-5 was expressed in all examined tissues, showing a significantly higher expression level in the cerebral ganglion (Figure 5). On the contrary, higher expression levels of IGFBP-5 in *P. fucata* and *M. yessoensis* have been documented in foot and mantle, respectively [9,20].

Expression patterns of Hdh IGFBP-5 mRNA in gonads at different stages of the gametogenetic cycle were analyzed (Figure 6). Differential expression was detected in different stages of the reproductive cycle, implying that the transcripts might be involved in reproductive regulation of Pacific abalone. In rainbow trout, IGFBP-5 mRNA is expressed in the ovary during the reproductive cycle, suggesting that this gene may play a role in reproduction by regulating IGFs [30].

The relative mRNA abundance of Hdh IGFBP-5 was highest in the polar body and cleavage stage, which might result from the maternal transcripts and involvement in the early development of *H. discus hannai* embryo. A similar pattern of expression was also observed for the *P. fucata* IGFBP [55]. Fan et al. [25] reported that Pf-IGFBP-5 was expressed in all examined developmental stages with the highest level in trochophore larvae.

Distribution of IGFBP has been reported in humans [56], rats [57], and zebra fish [58] using in situ hybridization. Distribution of IGFBP-5 mRNA has been detected in the mantle and pearl sac of *P. fucata* [25]. To date, no published reports are available on cellular localization of IGFBP-5 mRNA transcript in abalone tissues using in situ hybridization. In the present study, in situ hybridization revealed that this gene was expressed in the neurosecretory cells of the cerebral ganglion (Figure 8).

## 5. Conclusions

Our study represents the first report describing the structure and expression pattern of IGFBP-5 in *H. discus hannai*. The analysis of the phylogenetic tree and structural profiles of Hdh IGFBP-5 will aid in better understanding the IGFBP family evolution in vertebrates and invertebrates. Results of the in situ hybridization assay indicated that Hdh IGFBP-5 might be synthesized from neural ganglia. The expression pattern of gonads during the reproductive cycle and early developmental stages suggests that Hdh IGFBP-5 may play a role in reproductive regulation and ontogenic development by modulating the action of IGFs. The results of this study lay a foundation for further study on neuro-endocrinological regulation of IGFBP-5 in abalone.

## Supplementary Materials

The following are available online at https://www.mdpi.com/2079-7737/9/8/216/s1, Figure S1: Hdh IGFBP-5 mRNA expression in the neural ganglion (cerebral, pleuropedal, and branchial), adductor muscle, mantle, and gonad (testis and ovary) was determined by semi-quantitative RT-PCR. For normalization of mRNA expression in tissues, RPL-5 gene was used as an internal reference.
