# Supplementary material for: Characterization of Insulin-Like Growth Factor Binding Protein-5 (IGFBP-5) Gene and Its Potential Roles in Ontogenesis in the Pacific Abalone, Haliotis discus hannai
Source: Biology (Basel). 2020 Aug 9;9(8):216. doi: 10.3390/biology9080216 (PMC7465962; doi:10.3390/biology9080216)
Supplement: Supplementary file 1 [file biology-09-00216-s001.pdf]

**Supplementary figure**

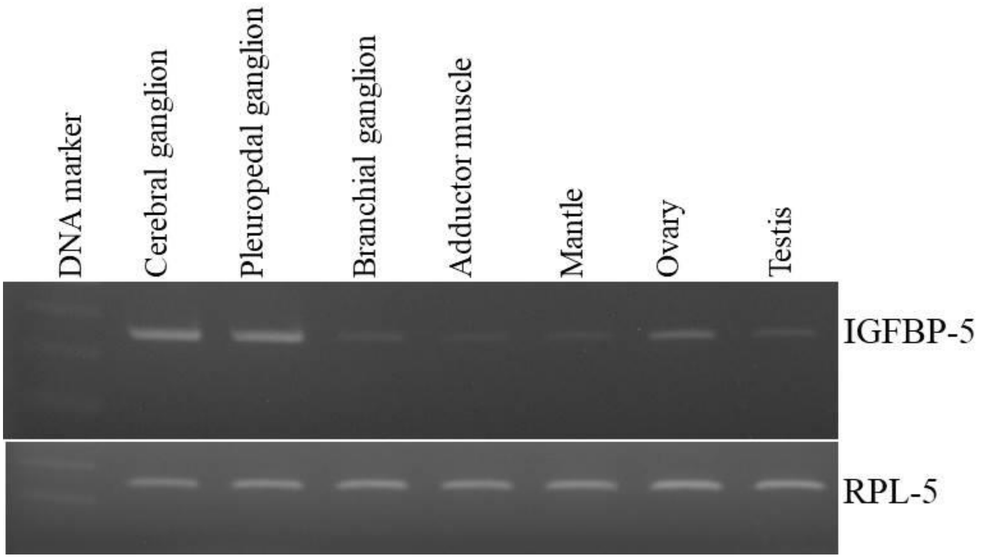


**Figure S1.** Hdh IGFBP-5 mRNA expression in the neural ganglion (cerebral, pleuropedal, and branchial), adductor muscle, mantle, and gonad (testis and ovary) was determined by semi-quantitative RT-PCR. For normalization of mRNA expression in tissues, RPL-5 gene was used as an internal reference.
